# Applying the UTAUT2 Model to Smart Eyeglasses to Detect and Prevent Falls Among Older Adults and Examination of Associations With Fall-Related Functional Physical Capacities: Survey Study

**DOI:** 10.2196/41220

**Published:** 2023-05-12

**Authors:** Justine Hellec, Meggy Hayotte, Frédéric Chorin, Serge S Colson, Fabienne d'Arripe-Longueville

**Affiliations:** 1 Université Côte d'Azur LAMHESS France; 2 Ellcie Healthy Antibes France; 3 Université Côte d'Azur CHU France

**Keywords:** Unified Theory of Acceptance and Use of Technology 2, fall prevention, fall detection, older people, older adults, facilitating conditions, effort expectancy, smart eyeglasses

## Abstract

**Background:**

As people age, their physical capacities (eg, walking and balance) decline and the risk of falling rises. Yet, classic fall detection devices are poorly accepted by older adults. Because they often wear eyeglasses as they go about their daily activities, daily monitoring to detect and prevent falls with smart eyeglasses might be more easily accepted.

**Objective:**

On the basis of the Unified Theory of Acceptance and Use of Technology 2 (UTAUT2), this study evaluated (1) the acceptability of smart eyeglasses for the detection and prevention of falls by older adults and (2) the associations with selected fall-related functional physical capacities.

**Methods:**

A total of 142 volunteer older adults (mean age 74.9 years, SD 6.5 years) completed the UTAUT2 questionnaire adapted for smart eyeglasses and then performed several physical tests: a unipodal balance test with eyes open and closed, a 10-m walk test, and a 6-minute walk test. An unsupervised analysis classified the participants into physical performance groups. Multivariate ANOVAs were performed to identify differences in acceptability constructs according to the performance group.

**Results:**

The UTAUT2 questionnaire adapted for eyeglasses presented good psychometric properties. Performance expectancy (β=.21, *P*=.005), social influence (β=.18, *P*=.007), facilitating conditions (β=.17, *P*=.04), and habit (β=.40, *P*<.001) were significant contributors to the behavioral intention to use smart eyeglasses (*R*²=0.73). The unsupervised analysis based on fall-related functional physical capacities created 3 groups of physical performance: low, intermediate, and high. Effort expectancy in the low performance group (mean 3.99, SD 1.46) was lower than that in the other 2 groups (ie, intermediate: mean 4.68, SD 1.23; high: mean 5.09, SD 1.41). Facilitating conditions in the high performance group (mean 5.39, SD 1.39) were higher than those in the other 2 groups (ie, low: mean 4.31, SD 1.68; intermediate: mean 4.66, SD 1.51).

**Conclusions:**

To our knowledge, this study is the first to examine the acceptability of smart eyeglasses in the context of fall detection and prevention in older adults and to associate acceptability with fall-related functional physical capacities. The older adults with higher physical performances, and possibly lower risks of falling, reported greater acceptability of smart eyeglasses for fall prevention and detection than their counterparts exhibiting low physical performances.

## Introduction

### Background

The number of older adults (ie, 65 years and older) is expected to increase in the coming years [1]. Aging is associated with sarcopenia [2,3] and dynapenia [4], both of which lead to a decrease in the physical capacities required to perform the activities of daily living. For example, advanced age has a negative impact on unipodal balance [5,6], walking speed [7], and distance covered [8,9]. Daily activities are therefore achieved more slowly in older adults than their healthy younger counterparts [10]. In addition to the decline in physical capacities, part of the aging process is the aging of sensory functions, particularly vision [11]. Declining visual acuity with age constrains older adults to wear corrective lenses to compensate for these deficits. A total of 9 of 10 people 50 years and older wear corrective eyeglasses [12], making them daily life objects for all those with visual deficits. This suggests that the recently developed connected eyeglasses, which include sensors for monitoring daily living activities, might be used for prevention or health promotion purposes in older adults. It therefore seems essential to evaluate how acceptable older adults find eyeglasses in the context of fall detection and prevention. This study was designed to evaluate whether older adults would accept this new device in their health monitoring and to determine whether fall-related functional physical capacities are associated with the acceptability of the eyeglasses.

### Fall Detection and Prevention

A common health risk in older adults is falling, which can lead to increased morbidity, mortality, and premature admission to nursing homes [13]. A person is considered a “faller” when he or she has had at least 1 fall in the past 12 months [14,15]. During clinical assessment, it is possible to determine whether a person is at risk of falling, especially with a unipodal balance test with eyes open and eyes closed [6], comfortable walking speed measured over 10 m [16], or distance covered during a 6-minute walk test (6MWT) [17]. A deterioration in at least one of these functional physical capacities (eg, balance, walking speed, or distance) can lead to a fall. Early detection of changes in functional physical capacities might signal an enhanced risk of falling and prompt an intervention to reduce the fall risk. However, most physical tests are usually performed in a laboratory setting and have little ecological validity because of the standardization of the instructions during the tests. Testing older people in ecological situations with the use of connected eyeglasses could be more representative of daily living activities.

Nowadays, people who have been identified as having a higher risk of falling (eg, deterioration in gait, walking speed, or balance) are usually equipped in daily life with sensors (eg, a watch or a necklace) to detect a fall and alert emergency services. These sensors are stigmatizing and are rarely worn, which reduces their effectiveness [18]. Only 26.1% of users (ie, 6/23 older adults) were satisfied with the fall detection technology versus 53.3% (8/15) of caregivers [18]. It was therefore important to create a fall detection and prevention tool incorporated into an everyday object, such as eyeglasses.

The eyeglasses presented in this study are innovative because an inertial measurement unit (ie, a 3D accelerometer, a 3D gyroscope, and a barometer) sensor is embedded in a temple of the eyeglasses [19,20]. The eyeglasses are connected to a mobile app on a smartphone (itself connected to the internet). Throughout the paper, they will be called smart eyeglasses. They are able to detect a fall using an algorithm that integrates data from the inertial measurement unit. When a fall occurs, an alert from the smart eyeglasses is transmitted to the cell phone through the mobile app to warn emergency services and designated caregivers. These smart eyeglasses can also prevent falls with another algorithm that analyzes the intraparticipant evolution of several functional physical capacities (eg, walking, sit-to-stand movement, and balance). Thus, as soon as a decline in these capacities is detected, caregivers are alerted. This algorithm is still under development, and its alert features will be at the choice of the person using the glasses.

### Acceptability of Smart Eyeglasses by Older Adults

The analysis of acceptability provides insight into why some devices are chosen, accepted, and used more than others [21]. Acceptability is often reduced to the assessment of satisfaction [18]. However, several theoretical models have been developed to define constructs predicting technology use. Currently, the Unified Theory of Acceptance and Use of Technology 2 (UTAUT2) [22] is one of the most comprehensive and parsimonious models, and it powers predictive models of behavioral intention to use technology [23,24]. The UTAUT2 is an extension of the UTAUT in the consumer context [22]. Performance expectancy, effort expectancy, social influence, facilitating conditions, hedonic motivation, price value, and habit are the key constructs that influence behavioral intention to use technology. The UTAUT2 constructs are defined as follows: (1) performance expectancy refers to “the degree to which using a technology will provide benefits to consumers in performing certain activities,” (2) effort expectancy refers to “the degree of ease associated with consumers’ use of technology,” (3) social influence refers to “the extent to which consumers perceive that important others (eg, family and friends) believe they should use a particular technology,” (4) facilitating conditions refers to “consumers’ perceptions of the resources and support available to perform a behavior,” (5) hedonic motivation refers to “the fun or pleasure derived from using a technology,” (6) price value refers to “consumers’ cognitive tradeoff between the perceived benefits of the applications and the monetary cost of using them,” (7) habit refers to “the extent to which an individual believes the behavior to be automatic,” and (8) behavioral intention represents the intention to use a particular technology [22].

We suggested that impaired functional physical capacities might signal an enhanced risk of falling, and smart eyeglasses are a dedicated tool to address this issue. Based on self-efficacy theory [25], physiological states are one of the main predictive factors of self-perceptions, notably in older adults [26]. In addition, in a systematic review of the acceptance of health-related technology, the authors analyzed the results based on the UTAUT2 constructs and stressed that “a major factor contributing to increased effort to use technology is impairment, both physical and cognitive” [27]. Thus, we assume that functional physical capacities, as an essential part of the smart eyeglasses, will be related to their acceptability, especially through the construct of effort expectancy.

### Aims of the Study

The objectives of this study were to measure the acceptability of smart eyeglasses for fall detection and prevention in older adults using the reliable UTAUT2 model and to examine the role of selected functional physical capacities that determine fall risk (eg, time maintained in unipodal balance, walking speed, and distance walked in 6 minutes). To our knowledge, no study has yet examined the associations between acceptability constructs and fall-related functional physical capacities, so the examination of these relationships will be exploratory.

## Methods

### Ethics Approval

The participants gave written informed consent before the testing session. This study was conducted according to the Declaration of Helsinki revised in 2013 and was approved by the South Mediterranean Protection of Persons Ethics Committee (registration number 2015-A01188-41). The data collected were anonymous. No financial compensation was offered to participants.

### Study Design and Procedure

**Figure 1:**
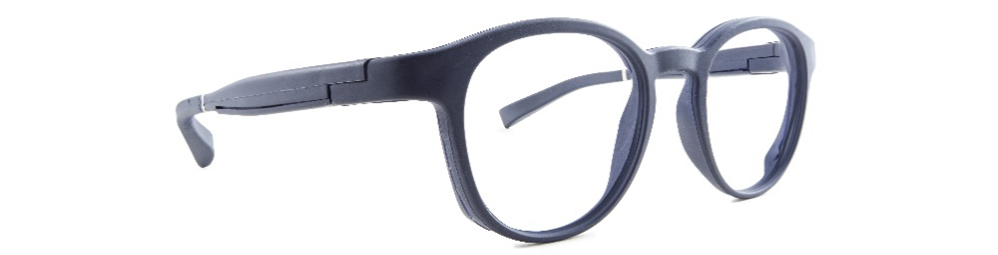
Smart eyeglasses (Ellcie Healthy) for fall detection and prevention in older adults.

The study was conducted on a frailty platform of a University Hospital Center in the south of France from October 2020 to the end of December 2021. A recruitment announcement was sent by email to all older adults affiliated with the “Office Municipal Niçois des Seniors.” To be included in this study, participants had to be (1) volunteers (ie, older adults who directly contacted the person in charge of the study), (2) 65 years or older, (3) able to understand and speak French, (4) independent in the activities of daily living, (5) able to walk without assistance, and (6) without knowledge of smart eyeglasses to detect and prevent falls (Figure 1). These inclusion criteria implied that the participants would not have a major psychiatric or neurological condition or orthopedic problems limiting their participation in activities of daily living. Participants could wear eyeglasses to correct their vision.

The single assessment consisted of a 1-hour session with several tests. First, the clinician collected the demographic information. Second, the smart eyeglasses were described orally in French to the participants. The following standardized information was delivered:

Smart connected eyeglasses look like a pair of classic eyeglasses with sensors embedded in the temples. Their objective is to take care of the wearer. Daily movements are measured with the smart eyeglasses and compared over time to assess risk and prevent falls. When a fall occurs, the eyeglasses send an alert to the older person’s relatives or to their health care provider/nurse in order to obtain help. Smart eyeglasses are connected to a cell phone with a mobile application that must be connected to the internet. The eyeglasses are a connected object, so they need to be charged every night with an adapted charger. The cost of the eyeglasses is 289 euros.

As the eyeglasses were being described, they were shown to the participants, who could try them, but they did not have to wear the eyeglasses at any time during the different tests. After this description, the participants completed the self-administered UTAUT2 questionnaire. Following the questionnaire, if the participants had any questions, they could ask them. Third, physical testing consisted of 3 assessments: unipodal balance with eyes open and closed, the 10-m walking test, and the 6MWT. Instructions for carrying out the tests and safety measures were explained to the participants before the tests were performed. The tests were performed in the same order for all participants.

### Measures

#### Acceptability of Smart Eyeglasses for Fall Prevention and Detection

To assess the acceptability of the smart eyeglasses for fall detection and prevention, the French UTAUT2 questionnaire [28] adapted for this specific technology was used. Thus, we replaced “*information and communication technology*” with “*eyeglasses for the detection and prevention of falls.*” Data were collected using a printed version of the adapted UTAUT2 questionnaire. It consisted of 25 items divided into 8 constructs: performance expectancy, effort expectancy, social influence, facilitating conditions, hedonic motivation, price value, habit, and behavioral intention. Each UTAUT2 construct is measured by 3 items, except for the construct of effort expectancy, which has 4 items. The UTAUT2 questionnaire was evaluated on a 7-point Likert scale where “1” means “strongly disagree” and “7” means “strongly agree.” The bifactor confirmatory model, as validated in the French validation article [28], provided a good fit to the data [29-32]: *χ*^2^/*df*=1.89, root mean square error of approximation=0.08, Tucker-Lewis index=0.90, and comparative fit index=0.93. Cronbach α ranged from .73 to .91, indicating good internal consistency [33].

#### Physical Tests to Assess the Risk of Falling

Three physical evaluations were performed to assess the risk of falling: unipodal balance in 2 conditions (ie, eyes open and eyes closed), the 10-m walking test, and the 6MWT. During the testing session, participants were barefoot, and tests were performed on a flat floor. The first physical test consisted of maintaining unipodal posture for the longest time possible in 2 conditions: eyes open and eyes closed. Participants were asked to lift their foot at the ankle level. The timer started as soon as the foot was off the ground and stopped as soon as the foot touched the ground. A stopwatch recorded the holding unipodal balance time for each foot (ie, left and right) for each condition in seconds. The average between the 2 sides was calculated for each test condition (ie, eyes open and closed) [34]. During the test, the clinician would stop the participant after 30 seconds of holding on one foot [5]. The second physical test consisted of walking for 10 m at a comfortable speed between the bars of the OptoGait system. The OptoGait software measured the spatiotemporal parameters of walking: step length, step time, speed, cadence, and distance. Here, we used the mean walking speed for 10 m, expressed in meters per second. The third physical assessment was the 6MWT, with the participants instructed to walk back and forth on the 10-m OptoGait system for 6 minutes, turning around immediately outside the bars. The test ended when the timer indicated 6 minutes. The total distance walked, expressed in meters, was extracted and recorded by the OptoGait software. Our population had a mean comfortable walking speed of 1.2 (SD 0.2) m/second, covered a mean distance of 390.2 (SD 75.2) m during the 6 minutes of walking, and maintained a mean of 19.0 (SD 10.6) seconds in unipodal balance with eyes open and 4.4 (SD 4.7) seconds with eyes closed, which is close to the usual values for healthy older adults [35-37].

### Statistical Analyses

Analyses were performed using the SPSS Statistics 23 software (IBM Corp), Amos v21 software (IBM Corp), and Python 3.6 (Python Software Foundation). The data sets were screened for missing values. The sample had no missing data, and the normality of the data was examined. The Shapiro-Wilk test showed that some variables did not follow a normal distribution. However, as this test was originally designed for samples smaller than 50 [38], we examined the kurtosis and skewness. The kurtosis and skewness were below the maxima recommended in the literature [39], so we concluded that the data were normal.

#### Associations Between Fall-Related Functional Physical Capacities and Acceptability of Smart Eyeglasses

Multiple regression analyses were used to examine the explained variance and the main contributors to behavioral intention to use the smart eyeglasses. Pearson bivariate correlations were computed to explore associations between the UTAUT2 constructs (ie, performance expectancy, effort expectancy, social influence, facilitating conditions, hedonic motivation, price value, habit, and behavioral intention) and functional physical capacities (ie, time maintained in unipodal balance with eyes open and closed, comfortable walking speed during the 10-m walking test, and distance covered during the 6MWT).

#### Clusters of Fall-Related Functional Physical Capacities

Generally, older people are categorized as fallers or not based on the response to a simple question: “Have you fallen at least once in the last 12 months?” If the participants answer yes, they are considered fallers, and if not, they are nonfallers. However, this definition is quite debated in the literature [40-42].

To better examine the possible associations between the constructs of the UTAUT2 and the 4 functional physical capacities tested, an unsupervised analysis was performed. Wong et al [43] used unsupervised analysis to determine fall risk groups based on fall-related functional physical capacities tests. In their unsupervised analysis, the K-means method was used to determine the fall groups and the optimal number of groups was established by the elbow method [44]. Participants were assigned to a group based on their common performance points. Similarly, in this study, we used an unsupervised analysis (K-means) to cluster participants into physical performance groups based on the functional physical capacities assessed. The optimal number of clusters was established with the elbow method. We labeled the groups regarding their characteristics of fall-related functional physical capacities.

#### Associations Between Clusters of Fall-Related Functional Physical Capacities and Acceptability of Smart Eyeglasses

A 1-way ANOVA with post hoc Tukey tests was performed to assess whether age differed between the groups. This demographic parameter was important to analyze because it has been shown to be a moderator of acceptability. A 1-way ANOVA with post hoc Tukey tests was also performed to assess whether height, body mass, BMI, and fall-related functional physical capacities differed between the groups. Proportions of females and proportions of fallers between groups were compared using *χ*^2^ tests.

The association between the physical performance group and smart eyeglasses acceptability was assessed with a multivariate ANOVA. When a between-group difference (*P*<.05) was significant, a post hoc Tukey analysis was used to determine the differences between the 3 groups. This analysis allowed us to highlight the constructs that were associated with the physical performance groups.

## Results

### Demographic Information

In this study, 142 volunteer participants (102 women and 40 men) were recruited. The mean age was 74.9 (SD 6.5) years, the mean height was 165.2 (SD 8.3) cm, the mean body mass was 67.6 (SD 14.1) kg, and the mean BMI was 24.7 (SD 4.6) kg/m². Fallers made up 35.2% (50/142) of the sample. People were considered fallers when they had fallen at least once in the past 12 months.

### Regression Analysis of the UTAUT2 Constructs

In a first step, we included sex and age as control variables in the regression analysis. The results were not significant, so the multiple regression analyses were computed without control variables (see Table 1). Performance expectancy, social influence, facilitating conditions, and habit were significant contributors to behavioral intention to use the smart eyeglasses. The acceptability constructs were found to explain approximately 73% of the variance in behavioral intention. We also examined differences in the acceptability of the UTAUT2 constructs between fallers and nonfallers and found no differences.

**Table 1:** Regression analyses (N=142).

Construct	β weight	*t* (*df*)	*P* value^a^	*F* (*df*)	*P* value	*R*²
Behavioral intention				50.88 (7)	<.001	0.727
*Performance expectancy*	.21	2.86 (141)	*.005*			
Effort expectancy	.02	0.25 (141)	.81			
*Social influence*	.18	2.74 (141)	*.007*			
*Facilitating conditions*	.17	2.11 (141)	*.04*			
Hedonic motivation	.06	0.94 (141)	.35			
Price value	–.03	–0.54 (141)	.59			
*Habit*	.40	4.71 (141)	*<.001*			

^a^Italicized constructs significantly explained behavioral intention.

### Associations Between Fall-Related Functional Physical Capacities and Acceptability of Smart Eyeglasses

No significant correlations were observed between performance expectancy, social influence, hedonic motivation, price value, habit, behavioral intention, and the 4 functional physical capacities. Significant small-to-moderate positive correlations were identified between effort expectancy and the physical variables: unipodal balance with eyes open (*r*=0.35, *P*<.001), unipodal balance with eyes closed (*r*=0.23, *P*=.007), walking speed (*r*=0.30, *P*<.001), and walking distance (*r*=0.33, *P*<.001). In addition, small significant positive relationships were found between facilitating conditions and the 4 functional physical capacities: unipodal balance with eyes open (*r*=0.25, *P*=.003), unipodal balance with eyes closed (*r*=0.21, *P*=.01), walking speed (*r*=0.29, *P*<.001), and walking distance (*r*=0.30, *P*<.001).

### Clusters of Fall-Related Functional Physical Capacities

**Figure 2:**
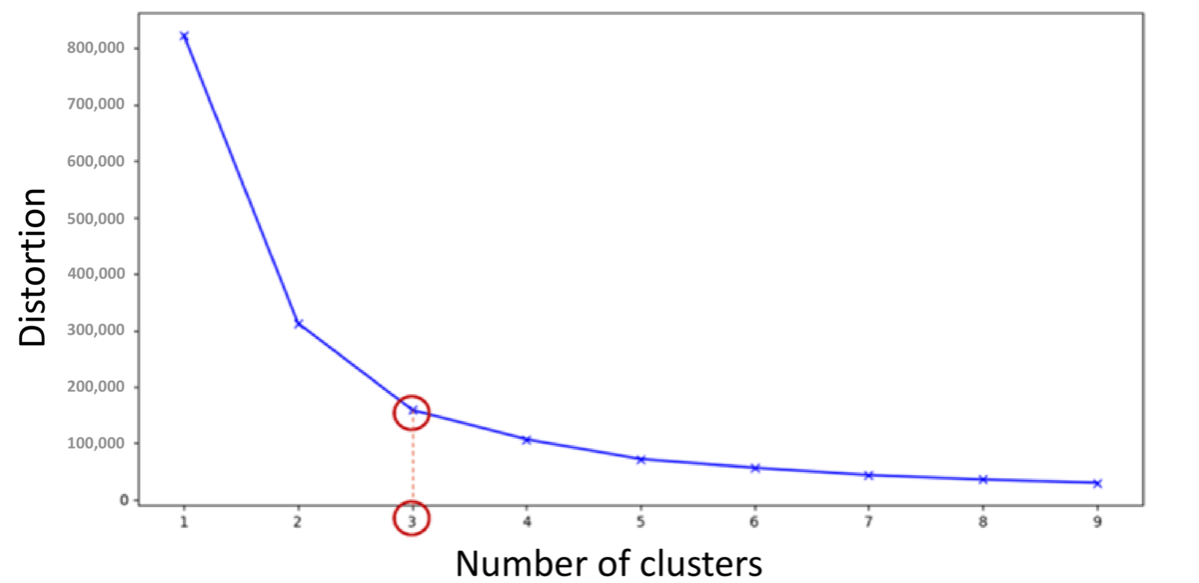
Elbow method showing the optimal number of clusters.

We obtained an optimal number of 3 clusters of the participants for the functional physical capacities tested (Figure 2).

The clustering algorithm classified the participants into 3 distinct groups. We labeled them as (1) low physical performance (ie, participants with the lowest functional physical capacities), (2) intermediate physical performance (ie, participants with the average functional physical capacities), and (3) high physical performance (ie, participants with the highest functional physical capacities). The physical performance of the participants in the low performance group was below the usual values reported for healthy older adults and close to those of frail older adults [16,45,46]. In contrast, the older adults in the high physical performance group exhibited physical performances above the usual values previously reported [45-47]. The means and SDs of the age, height, body mass, and functional physical capacities of the 3 groups, as well as the percentage of participants considered as fallers in each group (ie, based on the simple question: “Have you fallen at least once in the last 12 months?”), are reported in Table 2.

**Table 2:** Demographic data, functional physical capacities, and percentage of fallers of the 3 physical performance groups.

	Low performance group	Intermediate performance group	High performance group
Age (years), mean (SD)	78.63 (5.62)^a,b^	75.75 (6.05)^c^	70.65 (5.32)
Height (cm), mean (SD)	165.07 (8.20)	163.66 (7.92)	167.33 (8.45)
Body mass (kg), mean (SD)	67.28 (16.15)	68.11 (13.31)	67.09 (13.40)
BMI (kg/m²), mean (SD)	24.63 (5.35)	25.40 (4.45)	23.86 (3.94)
Sex: women, n (%)	25 (65.79)	47 (79.66)	30 (66.67)
Unipodal balance with eyes open (seconds), mean (SD)	9.49 (7.46)^a,b^	19.57 (10.26)^c^	26.33 (6.32)
Unipodal balance with eyes closed (seconds), mean (SD)	1.81 (1.85)^a,b^	4.13 (3.57)^c^	7.07 (6.12)
Walking speed (m/second), mean (SD)	1.03 (0.15)^a,b^	1.23 (0.15)^c^	1.37 (0.15)
Distance covered in 6 minutes (m), mean (SD)	293.90 (43.41)^a,b^	389.50 (21.47)^c^	472.34 (32.25)
Fallers, n (%)	14 (36.84)	21 (35.59)	15 (33.33)

^a^Significant difference between the low physical performance and intermediate physical performance groups.

^b^Significant difference between the low physical performance and high physical performance groups.

^c^Significant difference between the intermediate physical performance and high physical performance groups.

### Associations Between Clusters of Fall-Related Functional Physical Capacities and Acceptability of Smart Eyeglasses

Age significantly differed between the 3 performance groups (*F*_2,139_=21.18, *P*<.001). The low performance group (mean 78.63, SD 5.62 years) was older than the intermediate performance group (mean 75.75, SD 6.05 years, *P*<.001) and the high performance group (mean 70.65, SD 5.32 years, *P*<.001). The intermediate performance group was older than the high performance group (*P*=.02). There was a statistically significant difference in UTAUT2 constructs based on the performance group (*F*_8,264_=2.02, *P*=.01; Wilks Λ=0.79, partial η^2^=0.11). For each group, the means and SDs of each UTAUT2 construct are presented in Table 3.

Effort expectancy significantly differed between performance groups: *F*_2,139_=7.02, *P*=.001. Effort expectancy in the low physical performance group (mean 3.99, SD 1.46) was lower than that in the intermediate physical performance group (mean 4.68, SD 1.23; *P*=.04) and the high physical performance group (mean 5.09*,* SD 1.41; *P*<.001). Intermediate and high performance groups were not significantly different (*P*=.27).

For the facilitating conditions construct, performance groups were different: *F*_2,139_=5.68, *P*=.004. Facilitating conditions in the high physical performance group (mean 5.39, SD 1.39) was higher than those in the intermediate physical performance group (mean 4.66, SD 1.51; *P*=.04) and the low physical performance group (mean 4.31, SD 1.68; *P*<.001). The low and the intermediate performance groups presented no significant difference (*P*=.52).

For the other constructs, no significant differences were obtained—performance expectancy: *F*_2,139_=0.11, *P*=.89; social influence: *F*_2,139_=0.02, *P*=.98; hedonic motivation: *F*_2,139_=1.02, *P*=.36; price value: *F*_2,139_=0.77, *P*=.47; habit: *F*_2,139_=0.86, *P*=.43; and behavioral intention: *F*_2,139_=0.60, *P*=.55.

**Table 3:** Average ratings of the Unified Theory of Acceptance and Use of Technology 2 (UTAUT2) constructs for the 3 physical performance groups.^a^

UTAUT2 constructs	Low performance group	Intermediate performance group	High performance group
Performance expectancy, mean (SD)	3.89 (1.25)	3.78 (1.36)	3.75 (1.50)
Effort expectancy, mean (SD)	3.99 (1.46)^b,c^	4.68 (1.23)	5.09 (1.41)
Social influence, mean (SD)	3.68 (1.54)	3.66 (1.54)	3.72 (1.81)
Facilitating conditions, mean (SD)	4.31 (1.68)^c^	4.66 (1.51)	5.39 (1.39)^d^
Hedonic motivation, mean (SD)	3.82 (1.45)	4.21 (1.33)	4.14 (1.40)
Price value, mean (SD)	4.44 (1.53)	4.18 ±1.24)	4.47 (1.23)
Habit, mean (SD)	3.71 (1.38)	3.77 (1.42)	4.07 (1.42)
Behavioral intention, mean (SD)	4.30 (1.40)	4.12 (1.44)	4.44 (1.62)

^a^Value 1 represents “strongly disagree” and 7 indicates “strongly agree.”

^b^Significant difference between the low and intermediate physical performance groups.

^c^Significant difference between the low and high physical performance groups.

^d^Significant difference between the intermediate and high physical performance groups.

## Discussion

### Principal Findings

The objectives of this study were (1) to measure the acceptability of smart eyeglasses for fall detection and prevention in older adults using the UTAUT2 model and (2) to analyze the associations between the functional physical capacities that determine fall risk (eg, time maintained in unipodal balance, walking speed, and distance covered in 6 minutes) and the acceptability of these eyeglasses. Our results showed the moderate acceptability of the smart eyeglasses by older adults. Although the eyeglasses have some limitations, such as a limited battery life, the acceptability of the smart eyeglasses in our study seems to be higher than the acceptability of fall detection collars and smartwatches previously reported in the literature [18], which is promising for their commercialization.

The explained variance of behavioral intention to use the smart eyeglasses was 73%, with performance expectancy (β=.21, *P*=.005), social influence (β=.18, *P*=.007), facilitating conditions (β=.17, *P*=.04), and habit (β=.40, *P*<.001) as significant contributors. This result is consistent with the original UTAUT2 model, in which the explained variance was 74% [22]. These findings are also consistent with earlier studies showing that the UTAUT2 constructs are not always the predictors of behavioral intention, as this may vary with the technology under study [48]. The predictors of behavioral intention to use smart eyeglasses found in this study were not surprising. In light of the acceptability of the smart eyeglasses, performance expectancy can be taken to indicate the benefits of smart eyeglasses for fall detection and prevention in older adults, as previously reported in similar studies [49]. Social influence is a common factor of adherence to technology-based fall prevention programs in older adults [50,51]. Facilitating conditions are often reported as contributing to consumer acceptance and use of IT [48]. Habit has been shown to be a major determinant of behavioral intention, especially for new technologies [48,50,52]. The findings of this study also indicate that effort expectancy, hedonic motivation, and price value were not significant in explaining behavioral intention. The smart eyeglasses cost about the same as nonconnected eyeglass frames, and they do not incorporate any gamification strategies, which would explain that price value and hedonic motivation were not significant predictors. For effort expectancy, which refers to the degree of ease in using the smart eyeglasses, the literature is quite inconsistent. Although some studies report effort expectancy as a significant contributor to the behavioral intention to use therapeutic stepping exergames in older adults [49], other studies report that effort expectancy was not significant in explaining the behavioral intention to use mHealth in older adults [53]. One explanation for this lack of relationship between effort expectancy and behavioral intention was that a family member assisted older adults in using the technology [53]. In our context, the use of smart eyeglasses can also be assisted by a family member. For example, an older adult might wear the glasses but not use the app. Thus, the use of the device is not directly related to the use by an older adult him or herself.

The main originality of our study was to examine the associations between fall-related functional physical capacities and acceptability of the smart eyeglasses by older adults. To classify the population into physical performance groups, we used an unsupervised analysis (ie, K-means) based on the functional physical capacities assessed (ie, time maintained in unipodal balance, walking speed, and distance covered in 6 minutes), resulting in 3 fall-related physical performance groups. Two constructs of the UTAUT2 (ie, effort expectancy and facilitating conditions) were found to vary across the 3 groups, whereas no differences emerged for the other UTAUT2 constructs and behavioral intention. First, effort expectancy (ie, the degree to which participants believe that the technological device will be easy to use to monitor daily movements for the purpose of fall detection and prevention) was lower for the older adults in the low physical performance group compared with the other groups. This means that older adults with the lowest physical functional capacities perceived the smart eyeglasses as more difficult to use than their counterparts with higher physical performance. Second, facilitating conditions (ie, perceptions of the resources and support available to use the device) were higher in the high physical performance group (ie, with the lowest percentage of fallers) than those in the other 2 groups. Together these results suggest that the physical performance profile of older adults influences the perceived difficulty of using smart eyeglasses, as well as the available resources to use them, but not the intention to use them. Our findings are partly in line with the study of Keränen et al [54] showing that frailty or prefrailty was a significant predictor of the usefulness or usability of information and communication technologies among older adults.

This study has several strengths and suggests some practical implications. Several studies have proposed extensions of the UTAUT2 [27]. Some authors have associated the acceptability of new technologies with the cognitive capacities of older adults but not with functional physical capacities, at least to date [45]. To the best of our knowledge (eg, [50]), this study is the first to associate the UTAUT2 constructs with the physical capacities of older adults. As we showed that the youngest of our older adults reported the highest acceptability rates, this population could be targeted for the use of smart eyeglasses. Because habit was shown to be a major predictor of behavioral intention, it would be interesting to promote the use of similar technologies to form habits. Another perspective to increase acceptability, and specifically performance expectancy, would be to propose training workshops on smart eyeglasses.

Despite the many strengths of this study, some limitations must be noted. Smart eyeglasses were described orally to the participants, and they were told that technical assistance was available. Although this description is consistent with real-world conditions of use, it may have led to an overestimation of the facilitating conditions scores. As the participants were not aware of the device functionalities because of its novelty, it was difficult for them to know what their relatives thought about it, and this may have influenced social influence scores. Although female participants are generally more present in studies of fall prevention [55], the low participation of males in this study may limit the generalizability of the results. Although it was not assessed here, older adults with cognitive impairments often show low acceptability of technology [56], so it would be interesting in future work to evaluate the cognitive resources of older adults.

### Conclusions

To our knowledge, this study is the first to examine the acceptability of smart eyeglasses in the context of fall detection and prevention in older adults and to associate acceptability with fall-related functional physical capacities. The older adults in the low physical performance group (ie, the oldest with possibly the highest risk of falling) perceived the eyeglasses as more difficult to use (ie, lower effort expectancy) than the higher performance groups. In contrast, the older adults in the high physical performance group (ie, the youngest with the possibly lower risk of falling) perceived more facilitating conditions to use the eyeglasses. This study suggests that the physical performance profile of older people influences specific dimensions of technology acceptability. In future studies, it would be helpful to evaluate the acceptability of smart eyeglasses in the youngest older adult population with a longitudinal study design and daily use monitoring. Another research avenue would be to examine the influence of an adapted physical activity program on smart eyeglasses acceptability.

## Abbreviations

6MWT: 6-minute walking test
UTAUT2: Unified Theory of Acceptance and Use of Technology 2

## Data Availability

Participants of this study did not agree to have their data shared publicly. Moreover, as the data sets contained health information, they are protected by French laws. The data that support the findings of this study are available from the corresponding author, JH, upon reasonable request.
